# Annotations

**Published:** 1892-11-12

**Authors:** 


### ANNOTATIONS.

**Where are the Glasgow Newspapers?**

Mr. Gladstone tells us that medicine is to play a leading part in the civilisation of the future. The Premier is, we believe, not very remotely descended from a West of Scotland family. What does he think, what can any person think, of the state of civilisation in Lanarkshire at the present moment, as revealed by the annual report of Dr. James McLintock, the plain-speaking medical officer of the county? With regard to the housing of the working classes, Dr. McLintock writes: "In many things Scotland, and especially the county of Lanark, can challenge comparison with the rest of the kingdom, but most assuredly the condition of the dwellings of the working classes, and particularly of the miners, is not one of them. I am bound to state that, with a considerable experience of the dwellings of the labouring classes, I have never seen anything so uniformly bad as the condition of the miner's cottage and its surroundings in the midland counties of Scotland." On the next page of his report Dr. McLintock deals with the ashpits and privies of his district. He says—and it is desirable to note the language he employs—"These blots upon the face of the county are in direct opposition to all that is involved in modern hygiene: in many instances they are admirably contrived for polluting the air and water and soil in their vicinity." The ashpits are made large in order that they may not require to be emptied more than twice or thrice a year, and they are left unroofed and open to the vision in order that rubbish and all manner of filth may the more readily be thrown into them. They seem to serve the purpose of "privies" as well as of ashpits. "The condition of some of these 'conveniences,'" says the doctor, "is not infrequently loathsome, and must lead to nuisance being committed in the fields and lanes, and even to the retention for some time of offensive matters in the house." Now this is hardly fit to print in a respectable journal. What kind of men, women, and children must they be who are content to wallow year after year in this horrible physical mire? One would think the Glasgow newspapers, which are for the most part so ably and energetically conducted, would send out special commissioners and make the whole community of Lanarkshire ashamed of itself.

**A Clearing-house for the Unemployed.**

It is as much a part of a rational system of hospital administration to prevent feeble health and active disease as to cure those ills, and hence our strenuous interest in the cause of the unemployed. The Rev. F. G. Hobbins, Vicar of Christ Church, Stepney, explains, in a letter to the *Standard*, how excellently the "Clearing-house" for the unemployed works in the East-end of London, at any rate, on a small scale. By this system the prosperous may effectually help the unprosperous, without even so much as seeing them; and the rich dwellers in the country and the seaside may be brought into practical, if indirect contact with the poor of the towns. The Vicar of Christ Church gives a concrete example; a rich lady at the seaside writes to the Committee at the Central Clearing-house and explains to what extent she is prepared to help cases of *bona fide* need; she also states what religious denomination, if any, she would prefer to entrust her money with for administration. The Committee of the Clearing-house send her the name of a man with a family who is a workman genuinely out of work. She sends the man, through the Committee, a sovereign for immediate needs, and a few shillings a week for rent, coal, and bread, until work is found. When the beneficiary is once more in work the flow of benevolence is stopped, so far as he is concerned; but only that it may be directed into other channels if the resources are not dried up. This is excellent so far as it goes. But we submit that a great effort should now be made to systematically bring into knowledge, and within the range of help, all the distressed of this vast metropolis, and we further contend that, if some such scheme as that of the "North Kensington Friendly Workers," which we set forth at some length last week, were put in operation, the Clearing-house system, described by Mr. Hobbins, would form an admirable adjunct. The Clearing-house method does not seem to be of itself sufficient. An army of friendly investigators, with competent classifiers and organisers, is needed, and this the adoption of the Friendly Workers' system would secure.

**Oxford Doings Extraordinary.**

About a year ago our Special Commissioner, after a visit to Oxford, was constrained to declare that its medical school, the Radcliffe Infirmary, was out of date, and a disgrace to the city and the University. The authorities confirmed this judgment by asking for £10,000. A further illustration of "the way they have at Oxford" in matters medical is furnished by a correspondence, recently printed, between Professor Burdon Sanderson, Waynflete Professor of Physiology, and Dr. F. A. Dixey, his teaching assistant. The correspondence shows (a) That the Linacre Professor held an opinion to the effect that men reading for science honours, but not studying medicine, ought not to be burdened with the work of human dissection; (b) that Professor Burdon Sanderson held a contrary opinion; (c) that Dr. A. F. Dixey, Professor Sanderson's assistant, agreed with the Linacre Professor, but disagreed with Professor Sanderson; and furthermore, that he ventured to implement his conviction by seconding a motion before the Board of Faculty to that effect. Whereupon, Professor Sanderson, with all due solemnity, peremptorily dismisses his offending assistant. This, notwithstanding that Dr. Dixey has held his present appointment for eight years, and that his action in seconding the motion in question practically amounts to nothing more than the expression of a pious opinion. So far so good; or, rather, so bad. But there is more to follow. At Oxford there is a good thing for notable science workers in the shape of a Radcliffe Travelling Fellowship. The lucky travelling Fellow receives £200 a year for three years. But this most desirable fellowship is hampered by one condition which may render it unattainable by any man of independent judgment and mind. The condition is that of satisfying the peculiar soul of Professor Sanderson. Will it be believed that for the giving of this valuable and entirely desirable fellowship there is one examiner in each subject and one only? And one of those single examiners is Professor Sanderson. Will Professor Sanderson deny that the fellowship was recently refused to a young first-class man who had not happened to please his professorship, and was actually not bestowed at all because there was no other worthy competitor? And yet that young man before being refused the Radcliffe Fellowship, came up to London, and in open competition against a large number of picked young scientists, carried off the University Scholarship at a large Metropolitan medical school in triumph. That is the way in which science is encouraged at Oxford! Is it not obvious that neither Professor Sanderson nor any other person should be a single examiner, or for practical purposes, have such irresponsible power in the disposal of the Radcliffe Fellowship? There should not be one examiner only in each subject, but two at least! To put the full power of disposing of a valuable fellowship every year into the hands of one single "Crusty Christopher," as Tennyson called Kit North, is as absurd as its results are disastrous.

